# JAK2 Negative Polycythemia Vera

**DOI:** 10.4103/0974-2727.72215

**Published:** 2010

**Authors:** J P Geetha, C A Arathi, M Shalini, A G Srinivasa Murthy

**Affiliations:** Department of Pathology, Sri Siddhartha Medical College, Tumkur, Karnataka, India; 1 Department of Internal Medicine, Sri Siddhartha Medical College, Tumkur, Karnataka, India

**Keywords:** *JAK 2 V617F* mutation, polycythemia vera, BCSH guidelines

## Abstract

Polycythemia vera (PV) is a stem cell disorder, characterized as a panhyperplastic, malignant, and neoplastic marrow disorder. Several reasons suggest that a mutation on the Janus kinase-2 gene (*JAK2*) is the most probable candidate gene involved in PV pathogenesis, as *JAK2* is directly involved in intracellular signaling, following its exposure to cytokines, to which PV progenitor cells display hypersensitivity. A recurrent unique acquired clonal mutation in *JAK2* was found in most patients with PV and other myeloproliferative diseases (MPDs). A female patient of age 50 years, presented with hemiplegia, diplopia, and had a consistent rise in hemoglobin and hematocrit. Serum Erythropoietin (Epo) was decreased. *JAK2* mutation analysis was found to be negative. A diagnosis of polycythemia vera was made on the basis of the British Committee for Standards in Hematology (BCSH) guidelines.

## INTRODUCTION

Polycythemia vera is a rare disorder, with a minimum incidence of 2.6 per 100000.[1] In early 2005, a novel Janus kinase 2 (*JAK2*) mutation was described in association with PV, Essential thrombocythemia (ET), and primary myelofibrosis. *JAK’s* are cytoplasmic tyrosine kinases that mediate signaling from cytokine receptors to the cell nucleus. Mutational frequencies using sensitive detection techniques on either peripheral blood or bone marrow are estimated at 95% for PV and greater than 50% for ET.[2] However, it is very clear that some patients with classical PV lack *JAK2V617F* mutation, while some patients with other MPDs such as Idiopathic Myelofibrosis (IMF) and ET also express the *JAK2V617F* mutation.[1] The molecular basis of *JAK2V617F* negative PV is unknown.[3] Here is a case report of *JAK2V617F* negative polycythemia vera.

## CASE REPORT

A female patient of age 50 years presented with hemiplegia to the medical ward along with visual disturbances. The patient had a history of generalized weakness since two years. There was no history of smoking or alcohol intake and the patient was not hypertensive. The respiratory system and cardiovascular system were normal. Previous investigations showed a consistent rise in hemoglobin and hematocrit, with normal total leucocyte count and platelet count. On examination, the patient had mild splenomegaly confirmed on sonography; laboratory investigation revealed Hb% 19.6 gm%, hematocrit 0.62, TLC 9,200 cells/cumm, and a platelet count of 3.29 lakhs/cmm. Coagulation assays were normal, kidney and liver function tests were normal, arterial blood gas analysis was normal, and bone marrow showed a normoblastic appearance, with slight erythroid dominance. Serum Epo level was 3.09 mIU/ml. CT and MRI of the brain revealed multiple white matter infarcts. Secondary causes of erythrocytosis were ruled out. Cytogenetic analysis for *JAK2V617* mutation was done, which was negative.

A diagnosis of polycythemia vera was made on the basis of the British Committee for Standards in Hematology (BCSH) guidelines. The discovery of *JAK2* mutation in the majority of patients with PV allowed revision and simplification of the diagnostic criteria.[4] These criteria were as given in Table 1. Diagnosis of PV was made as A1 + A2 + A3 and B3 + B4, as present in our case. The patient underwent phlebotomy and was put on hydroxyurea. The patient symptomatically responded well to the treatment.

**Table 1:** Diagnostic criteria for polycythemia vera[4]

**JAK2-positive polycythemia vera**
A1. High hematocrit (>0.52 in men, >0.48 in women)
or raised red cell mass (>25% above predicted)
A2. Mutation in JAK2
* Diagnosis require both criteria to be present*
**JAK2-negative polycythemia vera**
A1. Raised red cell mass (>25% above predicted)
or hematocrit ≥ 0.60 in men, ≥ 0.56 in women
A2. Absence of a mutation in JAK2
A3. No case of secondary erythocytosis
A4. Palpable splenomegaly
A5. Presence of an acquired genetic abnormality
(excluding BCR-ABL) in the hemotopoietic cells
B1. Thrombocytosis (platelet count >450 × 10^9^/l)
B2. Neutophil leucocytosis (neutrophil count >10 × 10^9^/l
In non smokers, >12.5 × 10^9^/l in smokers)
B3. Radiological evidence of splenomegaly
B4. Endogenous erythroid colonies or low serum erythropoietin
Diagnosis requires A1 + A2 + A3 + either one other A or two B criteria

## DISCUSSION

*JAK2* gene is located on the short arm of chromosome 9, and loss of heterozygosity on chromosome 9p due to uniparental disomy is the most common cytogenetic abnormality in PV.[5] Only a minority of sporadic patients with PV do not carry *JAK2*, and the proportion of familial cases of PV that are negative is definitely higher.[6]

The workup of a patient with suspected PV could be initiated with peripheral blood mutation screening for *JAK2V617F*. To minimize the consequence of false positive or false negative test results, as well as, to capture the few cases that are *JAK2V617F* negative PV, we recommended the concomitant measurement of serum Epo levels, which is abnormally low in more than 90% of patients with PV. If the results of both the tests are suggestive of PV, the diagnosis is likely; bone marrow examination is encouraged, but not essential for making the diagnosis.[2]

Diagnostic criteria for PV were originally devised by the Polycythemia vera study group in the USA, in 1960. They required increased red cell mass and additional diagnostic criteria. They also included tests that were obsolete, such as, increased leucocyte alkaline phosphate or unbound B12 binding capacity. The World Health Organization (WHO) introduced criteria for diagnosis in 2001. These accepted an increased red cell mass or an Hb above 18.5 g/dl in men or 16.5 g/dl in women, as well as additional criteria. These criteria were over-permissive, as they allowed a diagnosis with an Hb that would be considered within the normal range. Some of the criteria were tests that were only available in a few laboratories and were not validated, such as, endogenous erythroid colony formation. In the UK, Pearson defined criteria that were designed to include only patients who were likely to have the diagnosis and be able to make a diagnosis using tests that were widely available. These criteria were adapted in the British Committee for Standards in Hematology (BCSH) guidelines for diagnosis and management.[4] The discovery of JAK2 mutations in the majority of patients with PV simply showed the presence of clonal disease. This allowed for revision and simplification of the diagnostic criteria in those with a JAK2 mutation. The amendment to the guidelines for the diagnosis was adopted by the BCSH and the criteria are as shown in Table 1.[4,7] The WHO also revised their criteria in view of the presence of JAK2 mutations in the majority of patients.[4]

BCSH criteria are the most accurate diagnostic criteria for PV as they have an acceptable level of sensitivity and can differentiate between PV and other erythrocytosis.[8] In the newly proposed diagnostic criteria for PV, the presence of *JAK2V617F* mutation has been integrated as a major criterion. It has been suggested that *JAK2V617F* mutation analysis can be used to screen individuals with polycythemia.[9]

There is no significant difference in the clinical presentation of the *JAK2* mutation in positive and negative PV patients. In general, patients who do express *JAK2V617F* are older than those who do not, but they do not have a longer duration of disease.[5]

In recent times, mutations of *JAK2* exon 12 have been described in *JAK2V617F* negative patients with PV.[10] Most patients with PV, carrying an exon 12 mutation, had isolated erythrocytosis at clinical onset, unlike patients with *JAK2* positive PV, who had elevations in leucocytes and/ or platelet count.[6] Both leucocytes and platelet counts were within normal limits in the present case. A detection method for exon12 mutation is currently available at limited centers.

Of late, the Medical Research Council Primary Thrombocythemia-1 Trial studied the effect of *JAK2* mutation on the treatment outcome in PV and ET patients, demonstrating that *JAK2* mutation in positive patients was more sensitive to hydroxyurea and not to anagrelide, than in those without *JAK2* mutation. Furthermore, the rate of arterial thrombosis appeared to be lower in *JAK2* positive patients receiving hydroxyurea compared to those receiving anagrelide, an effect that was not evident in *JAK2* negative patients. These results support the concept of classifying PV or ET as *JAK2* positive or negative during diagnosis and designing treatment strategies.[9] This case is presented because of its rarity and to highlight the importance of JAK2 mutation in polycythemia vera.
